# Insulin Glargine and Acarbose in the treatment of elderly patients with diabetes

**DOI:** 10.12669/pjms.35.3.86

**Published:** 2019

**Authors:** Jing Li, Jinzhi Ji, Fuyan Liu, Lingling Wang

**Affiliations:** 1 *Jing Li General Medicine (I), Binzhou People’s Hospital, Shandong, 256610, China*; 2 *Jinzhi Ji Department of Health Management Center, Binzhou People’s Hospital, Shandong, 256610, China*; 3 *Fuyan Liu Department of Operation Room, Binzhou People’s Hospital, Shandong, 256610, China*; 4 *Lingling Wang General Medicine (I), Binzhou People’s Hospital, Shandong, 256610, China*

**Keywords:** Elderly diabetics, Insulin Glargine, Acarbose

## Abstract

**Objective:**

To investigate the clinical efficacy of insulin glargine combined with acarbose in the treatment of elderly patients with diabetes.

**Methods:**

One hundred and forty-four elderly patients with diabetes who received treatment between December 2016 and December 2017 in Binzhou People’s Hospital, China, were selected and divided into a control group and an observation group, 72 each, using random number table. The control group was treated with insulin glargine, while the observation group was treated with insulin glargine combined with acarbose. The therapeutic effect, improvement of quality of life and adverse reactions were compared between the two groups.

**Results:**

After treatment, fasting blood glucose (FBG), 2h postprandial blood glucose (PBG) and glycosylated hemoglobin (Hb Alc) of the two groups were lower than those before treatment, and the decrease degree of the observation group was significantly larger than that of the control group (P<0.05). The time needed for blood glucose reaching the standard level and daily insulin dosage of the observation group were significantly lower than that of the control group, and the differences were statistically significant (P<0.05). SF-36 scale score of the observation group was significantly better than the control group, and the difference was statistically significant (P<0.05). There was no significant difference in the incidence of adverse reactions between the two groups (P>0.05).

**Conclusion:**

The combination of insulin Glargine and Acarbose can significantly control the blood glucose level of elderly patients with diabetes, improve the biochemical indicators, and enhance the quality of life. It is worth promotion in clinical practice.

## INTRODUCTION

Diabetes, as a chronic endocrine disease, has a high incidence in the middle-aged and elderly population, seriously affecting the physical health and quality of life.^1^,^2^ In recent years, with the improvement of living standards and medical conditions, the number of aging population has increased, and the incidence of diabetes has also increased.

Pathogenic factors of diabetes include genetic factors, environmental factors and physiological aging, resulting in insulin secretion or utilization disorders.^3^ Diabetes may lead to a variety of cardiovascular diseases due to long-term impairment of glucose metabolism and high blood glucose level.^4^ In addition, poor long-term glycemic control often results in peripheral neuropathy. Poor glycemic control can also affect important organs, which will aggravate the deterioration of diabetic patients and even affect life safety.^5^

In the past, insulin therapy was the main therapy to effectively control blood glucose level and reduce complications. Because of the need for long-term use, the application of single drug may weaken the effect of treatment.^6^ Elderly patients have weak constitution and poor immunity, and most of them also suffer from chronic diseases such as hypertension or hyperlipidemia. In order to effectively control blood glucose level for a long time and reduce complications as far as possible, two drugs are combined together usually.^7^ In recent years, insulin combined with oral hypoglycemic drugs has been gradually used in clinical treatment. In this study, insulin glargine and insulin analogue acarbose were used in elderly patients with diabetes, and the effect of treatment was analyzed.

## METHODS

One hundred and forty-four elderly patients with diabetes who received treatment in our hospital in the period from December 2016 to December 2017 were selected and divided into an observation group (n = 72) and a control group (n = 72) using random number table. There were 40 males and 32 females in the control group, and they aged 62-75 years (average (68.41±4.46) years) and had a disease course of 2-12 years (average (8.86±2.35) years). There were 38 males and 34 females in the observation group, and they aged 61-77 years (average (68.92±4.75) years) and had a disease course of 2-14 years (average (9.04±3.78) years). There was no significant difference between the two groups in age, course of disease, gender and other clinical data (P>0.05). The study was approved by the ethics committee of our hospital, and all the selected patients signed informed consent.

### Inclusion and exclusion criteria

Inclusive criteria included satisfying the clinical diagnostic criteria of type 2 diabetes formulated by the Diabetes Society of Chinese Medical Association, over 60 years, and having good mental status. Exclusion criteria included having renal and liver dysfunction; having acute and chronic complications of diabetes, having severe hypertension and having allergic constitution or being allergic to the studied drugs.

Both groups received conventional treatment. On that basis, patients in the control group were subcutaneously injected with insulin glargine (Sanofi-Aventis Deutschland Gmb H, batch number: J2014052), 10 U each time, once a day, and the dose was adjusted according to the actual blood glucose level of patients. Patients in the observation group were given insulin glargine combined with acarbose (Hangzhou Huadong Medicine Co., Ltd., China; batch number: H20020202). The dosage of insulin glargine was the same with the control group. Acarbose was orally administrated, 50 mg each time, three times each day. The dose was gradually increased to 100 mg each time. Patients in both groups were treated for three months.

Two groups of patients received individualized nursing intervention during the treatment. Based on the actual condition of each patient, including the patient’s age, course of disease, psychological status and the severity of diabetes, the corresponding individualized nursing intervention scheme was formulated according to Chinese Guidelines for the Prevention and Treatment of Type 2 Diabetes Dietary Care.^8^ A diet plan was formulated according to the weight of elderly patients, and exercise was controlled. Patients weighed once a week. The weight of patients was controlled to close to the ideal weight through diet. Moreover the diet was balanced.

Sugar intake was controlled, more coarse grain was suggested. They were provided with more green vegetables and asked to correct bad eating habit and give up smoking and alcohol. Psychological nursing was also needed. Medical staffs frequently communicated with elderly patients to understand the actual psychological state of patients to ensure timely psychological intervention of patients. Health education was carried out. The medical staffs carried out diabetes health education to patients regularly. Patients understood the pathogenesis of diabetes, commonly used hypoglycemic agents and correct dosage time, site and dose of insulin through watching videos or reading cards. Sports nursing were provided. The elderly diabetic patients had regular exercise, and the medical staffs set the appropriate time of exercise according to their actual physical conditions. The last content was rehabilitation nursing. The medical and nursing staffs encouraged the elderly patients to take proper rehabilitation exercises according to their own conditions, nursing schemes and physical fitness, formulate long-term rehabilitation goals for patients, and guide them to exercise.

### Observational indicators

The blood glucose control of the two groups before and after treatment was recorded, and the pre-treatment and post-treatment levels of fasting blood glucose (FBG), two hour postprandial blood glucose (PBG) and glycosylated hemoglobin (Hb Alc), the time needed for reaching the standard level of blood glucose and the daily insulin dosage of the two groups were analyzed and compared. The quality of life of the two groups was evaluated by SF-36 scale and compared before and after treatment. The total score was 100 points; the higher the score, the better the quality of life. The occurrence of adverse reactions of the two groups were observed and compared.

### Statistical analysis

The data were processed using SPSS 21.0. Measurement data were expressed by mean±SD and processed by t test. Enumeration data were expressed by n (%) and processed by Chi-square test. Difference was considered as statistical significant if P<0.05.

## RESULTS

The levels of FBG, 2h PBG and Hb Alc of the two groups had no significant differences before treatment (P>0.05) and decreased significantly after treatment; the decrease of the observation group was more obvious. The differences between the two groups after treatment had statistical significance (P<0.05, (Table-I).

**Table-I:** Blood glucose control effect between the two groups.

Group		FBG (mmol/L)	2hPBG (mmol/L)	Hb Alc (%)
Observation group	Before treatment	12.27±2.75	15.77±3.43	13.67±2.46
	After treatment	7.26±2.03^*#^	8.26±2.11^*#^	4.73±1.96^*#^
Control group	Before treatment	12.39±2.82	15.61±3.32	13.72±2.37
	After treatment	9.93±1.86^*^	10.79±2.33^*^	7.18±2.03^*^

* Note: indicated P<0.05 comparing to before treatment;

# Indicated P<0.05 comparing to the control group.

The time needed for reaching the standard level of blood glucose and daily insulin dosage in the observation group were significantly lower than those in the control group (P<0.05, Table-II). There was no significant difference in SF-36 score between the observation group and the control group before treatment ((53.132±4.43) vs. (53.132±4.43)) (t=0.814, P>0.05). After treatment, the SF-36 score of the observation group was (81.23±5.64), which was significantly higher than that of the control group (72.57±5.35), and the difference was statistically significant (t=7.217, P<0.05). After treatment, the SF-36 score of observation group and control group were higher than those before treatment, and the difference was statistically significant (t1=26.351, P1<0.05, t2 = 19.586, P2<0.05).

**Table-II:** Comparison of time needed for reaching the standard level of blood glucose and daily dosage of insulin between the two groups.

Group	Time needed for reaching the standard level of blood glucose (d)	Daily dosage of insulin (U)
Observation group	6.7±1.1	19.1±7.5
Control group	11.6±1.2	25.3±8.2
T	5.847	7.914
P	<0.05	<0.05

There were three cases of hypoglycemia and three cases of gastrointestinal reaction in the observation group (8.33%); there were four cases of hypoglycemia and 3 cases of gastrointestinal reaction in the control group (9.72%). There was no significant difference in the incidence of adverse reactions between the two groups (P>0.05).

## DISCUSSION

This study showed that insulin glargine combined with acarbose was effective in the treatment of elderly diabetic patients. Based on the analysis of the characteristics of elderly type 2 diabetes, it is concluded that elderly diabetes has a high incidence and a mild onset; therefore, it generally can not be taken seriously by patients. Thus the course of elderly diabetes is long, and most of the cases has cardiovascular diseases such as hypertension and coronary heart disease.^9^,^10^ Moreover elderly patients have poor physical quality and significantly decreased islet function; hence the effect of general drugs in controlling blood sugar is not ideal, and the effect of single drug treatment for senile diabetes is not obvious.^11^

Insulin glargine is a long-acting human insulin analogue produced by gene recombination technology and can simulate human basic insulin secretion.^12^ Its solubility in neutral solution is low. After subcutaneous injection, insulin glargine can be neutralized to form fine sediments which can steadily release insulin glargine to keep a stable blood concentration. The blood glucose concentration is maintained stable in that period, and there is no peak blood concentration.^13^,^14^ Numerous clinical studies have shown that insulin glargine therapy could effectively reduce the incidence of hypoglycemic events compared with premixed insulin and intermediate-acting insulin.^15^,^16^ Acarbose is a new type of hypoglycemic drug. After oral administration, it can inhibit the production of small intestinal α-glucosidase and prevent the decomposition of food polysaccharides to slowing down the absorption and utilization of glucose and reduce postprandial blood glucose level, especially for patients with normal fasting blood glucose but high postprandial blood glucose level.^17^-^19^

The results of this study showed that the blood glucose control effect of the observation group was significantly better than that of the control group, and the time needed for reaching the standard blood glucose level, daily insulin dosage and incidences of adverse reaction rate of the observation group were significantly lower than those of the control group (P<0.05), which was consistent with the research result of Qin.^20^ It indicated that the combination of the two drugs was more effective and stable in controlling blood glucose level than single drug and moreover was safe. The reason may be that insulin glargine focuses on maintaining the stability of blood glucose level within 24 hours, acarbose focuses on inhibiting the absorption and utilization of intestinal blood glucose after meals, and the combination of the two drugs can play a synergistic role in lowering blood glucose and controlling patients’ blood glucose level at a certain level throughout the whole day. The further exploration of quality of life suggested that the SF-36 scale score in the observation group was higher than that in the control group (P<0.05), indicating that insulin glargine combined with acarbose could help improve the quality of life and improve prognosis in the treatment of elderly diabetes.

## CONCLUSION

Insulin Glargine combined with Acarbose in the treatment of elderly patients with diabetes has a significant effect. It can significantly reduce blood sugar level, and improve the quality of life; hence it is worthy of promotion in clinical practice. The sample size was small in this study, and the long-term effect of blood glucose was not explored, which will be further studied by studies with a large sample size.

### Authors’ Contribution

**JL & JZJ:** Study design, data collection and analysis.

**FYL & LLW:** Manuscript preparation, drafting and revising.

**JL & LLW:** Review and final approval of manuscript.
